# Delivery of mGluR5 siRNAs by Iron Oxide Nanocages by Alternating Magnetic Fields for Blocking Proliferation of Metastatic Osteosarcoma Cells

**DOI:** 10.3390/ijms23147944

**Published:** 2022-07-19

**Authors:** Min A Kang, Pooja P. Rao, Hiroshi Matsui, Shahana S. Mahajan

**Affiliations:** 1 Ph.D. Program in Biochemistry, The Graduate Center of the City University of New York, 364 5th Ave., New York, NY 10016, USA; mkang@gradcenter.cuny.edu (M.A.K.); prao@gradcenter.cuny.edu (P.P.R.); hmatsui@hunter.cuny.edu (H.M.); 2 Department of Chemistry, Hunter College, City University of New York, 695 Park Ave., New York, NY 10065, USA; 3 Department of Medical Laboratory Science, Hunter College, City University of New York, 425 East 25th Street, New York, NY 10010, USA; 4 Ph.D. Program in Chemistry, The Graduate Center of the City University of New York, 364 5th Ave., New York, NY 10016, USA; 5 Department of Biochemistry, Weill Cornell Medical College, 413 East 69th Street, New York, NY 10021, USA; 6 Ph.D. Program in Biology, The Graduate Center of the City University of New York, 364 5th Ave., New York, NY 10016, USA; 7 Brain Mind Research Institute, Weill Cornell Medical College, 413 East 69th Street, New York, NY 10021, USA

**Keywords:** mGluR5 siRNA, iron oxide nanocages, LM7, OS482, alternative magnetic field

## Abstract

Although osteosarcoma is the most common primary malignant bone tumor, chemotherapeutic drugs and treatment have failed to increase the five-year survival rate over the last three decades. We previously demonstrated that type 5 metabotropic glutamate receptor, mGluR5, is required to proliferate metastatic osteosarcoma cells. In this work, we delivered mGluR5 siRNAs in vitro using superparamagnetic iron oxide nanocages (IO-nanocages) as delivery vehicles and applied alternating magnetic fields (AMFs) to improve mGluR5 siRNAs release. We observed functional outcomes when mGluR5 expression is silenced in human and mouse osteosarcoma cell lines. The results elucidated that the mGluR5 siRNAs were successfully delivered by IO-nanocages and their release was enhanced by AMFs, leading to mGluR5 silencing. Moreover, we observed that the proliferation of both human and mouse osteosarcoma cells decreased significantly when mGluR5 expression was silenced in the cells. This novel magnetic siRNA delivery methodology was capable of silencing mGluR5 expression significantly in osteosarcoma cell lines under the AMFs, and our data suggested that this method can be further used in future clinical applications in cancer therapy.

## 1. Introduction

Osteosarcoma is the most common primary bone malignancy that initially peaks in children and late adolescents [1,2]. Despite advances in modern medicine and treatment of osteosarcoma, the five-year survival rate has remained constant for over three decades [2,3] The major clinical issues in curing osteosarcoma are cancer recurrence and tumor progression due to the development of drug resistance in the tumor microenvironment [3,4,5,6,7]. A broad spectrum of chemotherapeutic drugs, including doxorubicin, cisplatin, methotrexate, and etoposide, are used to treat osteosarcoma; however, frequent development of multidrug resistance results in treatment failure [5,8]. Effective pharmaceutical treatments of osteosarcoma would be achieved if drug resistance is resolved and drugs can specifically target the tumor sites.

Recent studies have revealed that small interfering RNAs (siRNAs) play a critical role in the dysregulation of tumor progressions [9,10]. siRNAs are non-coding RNAs consisting of 20–25 nucleotides involved in the post-transcriptional regulation of gene expression by destabilizing mRNA transcription and suppressing its translation [11,12,13]. The stability of siRNAs, regulation of post-transcriptional gene expression, and ability to distinguish between normal and tumor tissues are potential advantages of using siRNAs as therapeutic agents [13,14]. Osteosarcoma is known to secrete glutamate and express glutamate metabolic receptors that involve autocrine/paracrine glutamate signaling to promote tumor growth [15,16,17]. Our previous studies have shown that type 5 metabotropic glutamate receptor (mGluR5) is expressed in LM7 cells. Knockdown of mGluR5 expression using Lentivirus ShRNA in the LM7 cells prohibited the formation of colonies, suggesting that mGluR5 plays an important role in glutamate-dependent proliferation of osteosarcoma cells [15,17,18]. Regulating mGluR5 expression in the cells that affect the tumor growth by delivering siRNA would be beneficial, since we can improve target-specific treatment and avoid cancer recurrence and tumor progression that is caused by drug resistance occurring in the tumor microenvironment. Therefore, in this work, we addressed the idea of silencing mGluR5 expression to inhibit cell proliferation by performing siRNA transfection.

In this report, to deliver mGluR5 siRNAs, we applied a magnetically driven delivery method using superparamagnetic iron oxide nanocages (IO-nanocages) as a carrier, which was previously investigated by Matsui’s group [19]. Magnetic nanoparticles have been used to develop biomedicine in cancer therapy, such as magnetic separation and therapeutic drug delivery [20]. Magnetic separators aggregate magnetically tagged biomaterials to separate them from unwanted supernatant by applying a high magnetic field gradient to capture them as they flow in the medium [21]. Cytotoxic drugs are often loaded on biocompatible magnetic nanoparticles to lower the cytotoxicity, and the high-gradient magnetic field is used when the complex is injected to concentrate the complex at the targeted sites [22,23]. Recently, Matsui’s group demonstrated that siRNA delivery efficiency was significantly enhanced when superparamagnetic IO-nanocages in a diameter of 20 nm were used to deliver siRNAs. As siRNAs-loaded IO-nanocages underwent Brownian relaxation in alternating magnetic fields (AMFs), this Brownian motion increased the endosomal escape and released more siRNAs in the cytoplasm, which enhanced the transfection efficiency [19]. A narrow hysteresis loop of DC magnetic saturation measurement with a superconducting quantum interference device (SQUID) confirmed that iron oxide nanoparticles are superparamagnetic, and these particles have negligible values of coercivity (remnant magnetization available after field is removed) [24,25,26,27]. The saturation magnetization of IO-nanocages with the magnetic relaxation time led to the recognition that AMFs in the certain frequency range could produce the Brownian motion of IO-nanocages that causes movement of nanoparticles to release loaded siRNAs. This magnetic Brownian motion was also observed to increase the endosomal escape and release siRNAs better in the cytoplasm, which further enhanced the transfection efficiency [19]. Therefore, we used the 20 nm IO-nanocages to deliver mGluR5 siRNAs, since these IO-nanocages could most effectively deliver siRNAs under AMFs.

Our goal was to deliver mGluR5 siRNAs using IO-nanocages in AMFs and induce the inhibition of the proliferation of osteosarcoma cells. We first studied the internalization pathways of IO-nanocages in LM7, metastatic human osteosarcoma, and OS482, metastatic mouse osteosarcoma cells. Then, we delivered firefly luciferase siRNA-loaded IO-nanocages in AMFs to luciferase-expressing LM7 and OS482 cells as a proof-of-concept. Then, we conducted the experiments to observe the change in mGluR5 expression in both cell lines by delivering mGluR5 siRNAs with IO-nanocages in the absence and presence of AMFs. Lastly, we analyzed the functional outcomes of silencing mGluR5 expression in LM7 and OS482 cell lines by performing MTT and clonogenic assays.

## 2. Results

### 2.1. Internalization of IO-Nanocages in LM7 and OS482 Cells

**Figure 1:**
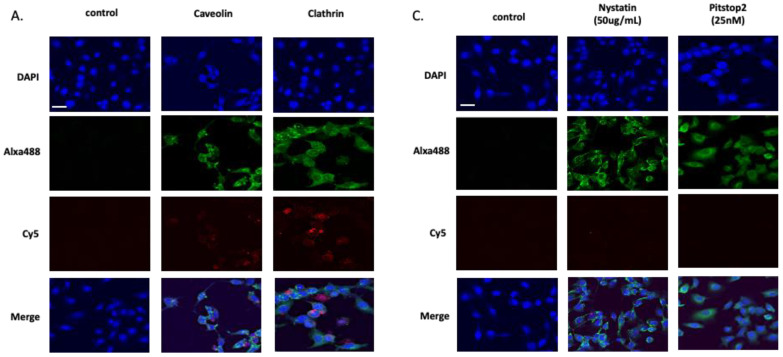

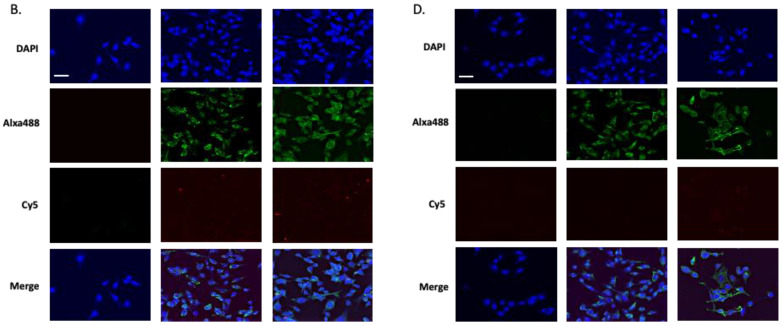
Confocal microscopy images of (**A**) LM7 human metastatic osteosarcoma cells and (**B**) OS482 mouse metastatic osteosarcoma cells after incubation with Cy5 labelled IO-nanocages for 6 h. (**C**) LM7 cells and (**D**) OS482 cells were pretreated with endocytosis inhibitors, nystatin (50 μg/mL) and Pitstop2 (25 μM) before incubation with Cy5 labelled IO-nanocages to evaluate the endocytic pathway of IO-nanocages in each cell line. Cell nuclei were stained with DAPI (blue), red represents Cy5 labelled IO-nanocages, and green indicates caveolin-1/Clathrin heavy chain antibody with Alaxa488 secondary antibody. The scale bar = 20 μm.

Clathrin and caveolae-receptor-mediated endocytosis are the most common pathways for nanoparticles to internalize into cells [28,29,30,31]. It has been shown that nanoparticles less than 50 nm in diameter have better clearance, biodistribution, and avoid phagocytosis better than larger nanoparticles [32,33,34]. Depending on the characteristics of the nanoparticles, such as size, shape, and surface charge, the endocytic pathways of the nanoparticles might be influenced [35,36,37]. Moreover, cell type, levels of receptor expression, and cell membrane elasticity would affect the endocytosis of the nanoparticles [35,38]. Therefore, we first studied which endocytic pathway is involved in LM7 and OS482 cells for the uptake of cage-shaped IO-nanocages with a size of 20 nm. Based on the assay, we confirmed that the clathrin and caveolin receptors were expressed in both LM7 and OS482 cells (Figure 1). Clathrin heavy chain and Caveolin-1, markers for clathrin and caveolae-mediated endocytosis, respectively, were observed in LM7 (Figure 1A) and OS482 cells (Figure 1B), and we confirmed the internalization of IO-nanocages in both cell lines.

To further characterize which pathway is predominant in each cell line, we applied Pitstop2 and nystatin to block the endocytic pathway when IO-nanocages are internalized. Pitstop2 is a cell membrane permeable clathrin inhibitor that disrupts the interaction of amphiphysin with the amino-terminal domain of clathrin, whereas nystatin is a cholesterol sequester that dissembles caveolae and cholesterol in the membrane [39,40,41]. Both LM7 and OS482 cells were cultured in DMEM with 10% FBS for 24 h and incubated in serum-free media for 1 h. As a following step, cells were pretreated with 25 μM Pitstop2 for 15 min and 50 μg/mL nystatin for 30 min individually at 37 °C before incubating with IO-nanocages.

Endocytosis of IO-nanocages was blocked in LM7 cells when cells were pretreated with Pitstop2, while nystatin did not completely block the internalization of IO-nanocages but reduced their internalization (Figure 1C). This result elucidated that IO-nanocages may enter LM7 cells via clathrin and caveolae-mediated endocytosis; however, they predominantly undergo the clathrin-mediated endocytosis. Endocytosis of IO-nanocages in OS482 cells showed a different pattern than in LM7 cells. We observed that the uptake of IO-nanocages was inhibited when nystatin was used for OS482 cells, while endocytosis was reduced when cells were pretreated with Pitstop2 (Figure 1D). IO-nanocages internalization was mainly mediated by caveolae involving endocytosis. Our findings suggested that the clathrin-mediated pathway plays a central role in the uptake of IO-nanocages in LM7 cells, whereas IO-nanocages were mainly endocytosed via the caveolae-mediated endocytosis pathway in OS482 cells.

### 2.2. Effect of the IO-Nanocages Delivery System in LM7 and OS482 Cells in the Absence and Presence of AMFs

**Figure 2:**
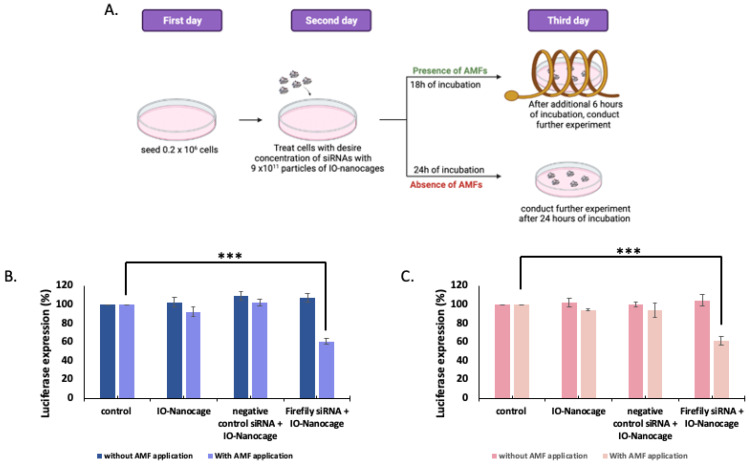
(**A**) Schematic image of siRNA delivery system experimental design using IO-nanocages in the absence and presence of AMFs. In vitro luciferase assay of firefly luciferase siRNA delivered by IO-nanocages in (**B**) luciferase-expressing LM7 human metastatic osteosarcoma cells, and (**C**) luciferase-expressing OS482 mouse metastatic osteosarcoma cells with and without application of AMFs (445 kHz). In both cell lines, firefly luciferase siRNA-loaded IO-nanocages under the AMFs showed the reduction of luciferase expression. *** *p* < 0.001, N = 4.

To examine the magnetically driven delivery system to transfect siRNAs, we delivered firefly luciferase siRNAs to luciferase-expressing LM7 and OS482 cells. Previously, Matsui’s group have shown that the internalization of IO-nanocages in the B16-F10 cells reaches saturation after 18 h of incubation, and the siRNA transfection efficiency improves significantly upon the application of AMFs [19]. Therefore, we examined the luciferase assay in the same experimental conditions as Matsui’s group examined previously. In this work, we treated the cells with siRNA-loaded IO-nanocages and applied AMFs after 18 h of incubation (Figure 2A). In the absence of AMFs, we treated the cells for 24 h and conducted further experiments.

The results showed that when firefly luciferase siRNAs were delivered with IO-nanocages under AMFs, the luciferase expression in both LM7 (Figure 2B) and OS482 (Figure 2C) cells was reduced. Luciferase expression of LM7 cells in this group was reduced to 60.7 ± 5.3%, while the luciferase expression in OS482 cells was decreased to 61.6 ± 4.8% when firefly siRNAs were delivered with IO-nanocages in AMFs. Based on the ANOVA single factor *t*-test, the *p*-value of the experimental group compared to the control (neat cells) was 4.45 × 10^−6^ for the LM7 cells and 2.54 × 10^−4^ for the OS482 cells, indicating a significant reduction in luciferase expression in the experimental groups compared to that of the control when the AMFs were applied. These results elaborated that luciferase expression decreased significantly when firefly luciferase siRNAs were delivered by IO-nanocages in the presence of AMFs and supported our hypothesis of enhanced transfection efficiency of mGluR5 siRNAs using this magnetic nanoparticle delivery system in osteosarcoma cells.

### 2.3. The Reduction of mGluR5 Expression by Delivering mGluR5 siRNA-Loaded IO-Nanocages under AMFs in Osteosarcoma Cells

**Figure 3:**
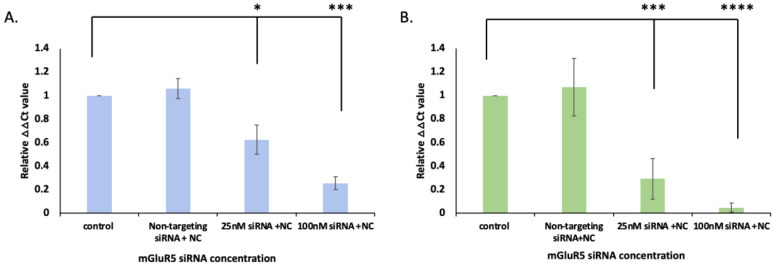
Quantitative RT-PCR was performed to detect the mGluR5 expression in (**A**) LM7 human metastatic osteosarcoma cells and (**B**) OS482 mouse metastatic osteosarcoma cells. When 100 nM mGluR5 siRNAs were loaded to IO-nanocages and delivered in both LM7 and OS482 cells, the mGluR5 expression was reduced significantly. The AMFs application was performed at 445 kHz. * *p* < 0.05, *** *p* < 0.001, **** *p* < 0.0001, N = 4.

To further confirm if the reduction of expression in cells is due to the delivery of siRNAs released from IO-nanocages under AMFs, we delivered mGluR5 siRNAs in both LM7 and OS482 cells and observed a change in mGluR5 expression. In Figure 3, the mGluR5 expression was quantified by quantitative RT-PCR before and after siRNA transfection, with respect to the concentration of mGluR5 siRNA 25 nM and 100 nM, to evaluate the transfection efficiency.

We used GAPDH and β-actin as the control to normalize mGluR5 expression in OS482 and LM7 cells, respectively. The results revealed that the higher reduction in mGluR5 expression occurred when the 100 nM of siRNA was incubated in both LM7 and OS482 cells (Figure 3). siRNA in 25 nM groups reduced the expression by 62.6 ± 12.5%, while the expression was decreased by 74.5 ± 5.5% when 100 nM of siRNA was incubated. This result showed a significant reduction in mGluR5 expression when 100 nM of siRNA was delivered, with a *p*-value of 1.08 × 10^−5^ compared to the control (neat cells) by ANOVA single factor *t*-test. mGluR5 expression in OS482 cells was also significantly decreased by 70.7 ± 17.2% and 95.4 ± 3.9% when 25 nM and 100 nM of siRNA were delivered under AMFs, respectively. The *p*-value of the experimental groups compared to the control revealed 4.65 × 10^−5^ and 4.17 × 10^−9^ for 25 nM and 100 nM of siRNA were delivered, respectively. These results indicated that both concentrations of siRNA delivered by IO-nanocages under AMFs significantly silenced the mGluR5 expression in OS482 cells, while the mGluR5 expression was significantly reduced in LM7 cells when 100 nM of siRNA was delivered by IO-nanocages with AMFs.

### 2.4. Silencing of mGluR5 Expression Leads to Inhibition of Cell Growth

**Figure 4:**
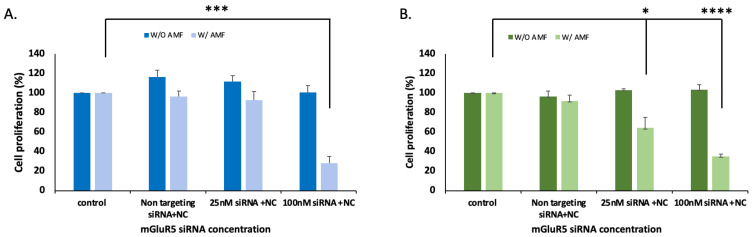
The proliferation (**A**) LM7 human metastatic osteosarcoma cells and (**B**) OS482 mouse metastatic osteosarcoma cells with and without AMF application (445 kHz) was measured by MTT assay. when 100 nM of mGluR5 siRNA was loaded to IO-nanocages and delivered under the AMFs, the cell proliferation was significantly decreased in both cell lines. * *p* < 0.05, *** *p* < 0.001, **** *p* < 0.0001, N = 3.

To assess the effect of the delivery of mGluR5 siRNA on cell growth, we performed MTT and clonogenic assays. We conducted the experiment by delivering mGluR5 siRNAs in two different concentrations with IO-nanocages in the absence and presence of AMFs. In the absence of AMFs, the proliferation of the LM7 cells in the control group (neat cells) was similar to the experimental groups (delivery siRNA in different concentrations with IO-nanocages). In contrast, the proliferation of cells decreased by 72.9 ± 7.2% when 100 nM siRNA was delivered, compared to the control group, in the presence of AMFs (Figure 4A). Moreover, the cell proliferation did not change in OS482 cells when mGluR5 siRNAs were delivered with IO-nanocages in the absence of AMFs compared to the control group. However, the proliferation of OS482 cells decreased by 36.8 ± 10.7% and 65.0 ± 2.3% when 25 nM and 100 nM mGluR5 siRNA were delivered with AMFs, respectively (Figure 4B). These results indicated that mGluR5 is a crucial receptor involved in the glutamate-dependent proliferation of LM7 and OS482 cells.

**Figure 5:**
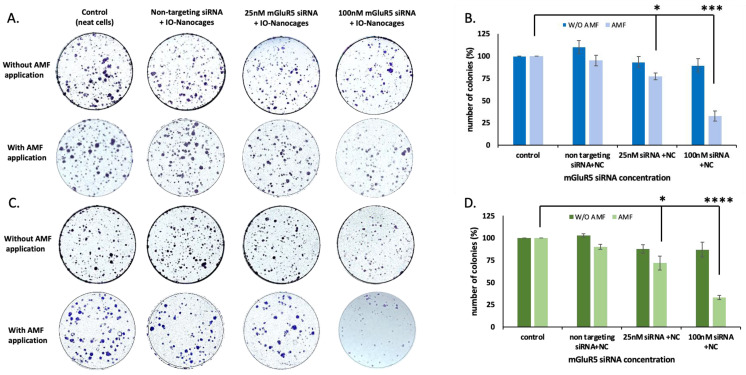
The colony formation of (**A**) LM7 human metastatic osteosarcoma cells and (**C**) OS482 mouse metastatic osteosarcoma cells with and without AMF application (445 kHz) was stained by 0.05% Cresyl violet. The number of colonies were quantified in (**B**) LM7 human metastatic osteosarcoma cells and (**D**) OS482 mouse metastatic osteosarcoma cells. When 100 nM of mGluR5 siRNA was incorporated to IO-nanocages with AMFs, the colony formation was significantly prohibited. * *p* < 0.05, *** *p* < 0.001, **** *p* < 0.0001, N = 3.

We further examined the ability of cell growth when mGluR5 expression was silenced by performing the clonogenic assay. Both LM7 and OS482 cells were grown in for three days, and cells were treated with mGluR5 siRNA-loaded IO-nanocages on the fourth day. After 18 h of treatment, AMFs were applied, and cells were fixed and stained with 0.05% Cresyl violet after an additional 6 h of AMFs application. In the absence of AMFs, the cells were fixed and stained after 24 h of treatment. We observed a decrease in colony formation by 23.7 ± 3.8% and 67.5 ± 5.6% when 25 nM and 100 nM of mGluR5 siRNAs, respectively, were delivered with IO-nanocages in the presence of AMFs (Figure 5A,B). This result revealed a significant inhibition of colony formation when 25 nM and 100 nM of siRNA was delivered, with a *p*-value of 0.0067 and 0.00048, respectively, compared to the control (neat cells) by ANOVA single factor *t*-test. Moreover, colony formation in OS482 cells was also significantly decreased by 28.0 ± 7.9% and 66.9 ± 2.4% when 25 nM and 100 nM of siRNA, respectively, were delivered with IO-nanocages under AMFs (Figure 5C,D). The *p*-value of the experimental groups to control revealed 0.037 when 25 nM of mGluR5 siRNAs was delivered and 1.77 × 10^−5^ when 100 nM of mGluR5 siRNA were delivered with IO-nanocages in the presence of AMFs. These results supported that mGluR5 plays a pivotal role in glutamate-dependent proliferation in both LM7 and OS482 cells by showing inhibition of cell growth and colony formation when mGluR5 siRNAs were delivered by IO-nanocages with AMFs.

## 3. Discussion

Previously, it has been reported that osteosarcoma secretes glutamate and the metabotropic glutamate receptor (mGluR) is highly expressed in osteosarcoma [15,17,18,42]. mGluR5 is especially involved in glutamate signaling for cell growth; therefore, knockdown of mGluR5 expression in osteosarcoma cells can disrupt the proliferation of the cells [15,17,18,43]. In this study, we demonstrated the reduction of mGluR5 expression in osteosarcoma by delivering mGluR5 siRNAs via IO-nanocages vehicles, and we observed the inhibition of cell growth when mGluR5 expression was silenced as AMFs were applied.

**Figure 6:**
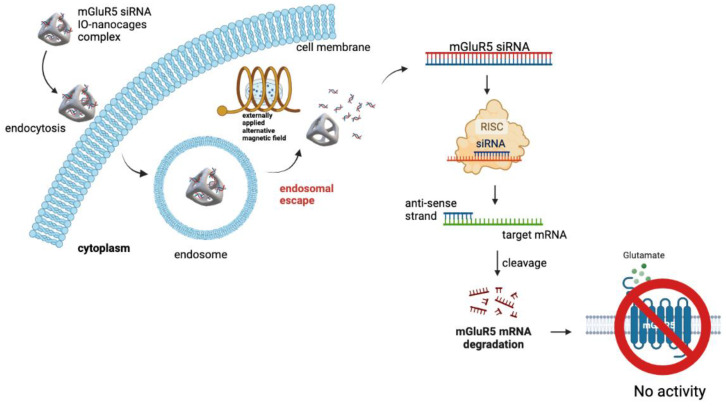
Schematic image of mGluR5 siRNA delivery system by using IO-nanocages in the presence of AMFs application. When mGluR5 siRNA releases in the cytoplasm after AMFs treatment, it will integrate with RNA-induced silencing complex (RISC) to undergo strand separation. Single strand within the complex will find its complementary mRNA, which result in mRNA degradation and prevent protein translation.

The delivery scheme is summarized in Figure 6. As we applied AMFs after siRNA-loaded IO-nanocages were endocytosed in LM7 and OS482 cells, the siRNAs released from the IO-nanocages interacted with RNA-induced silencing complex (RISC) in the cytoplasm. These siRNAs bind to its complementary mRNA to degrade mRNA and prevent mGluR5 protein expression [44,45,46]. Therefore, when a sufficient concentration of siRNAs is delivered to the cytoplasm, a significant enhancement of transfection efficiency can be accomplished. We confirmed that siRNAs were delivered more effectively with IO-nanocages when AMF was applied. Moreover, we found that cell proliferation and colony formation were significantly inhibited when mGluR5 expression was silenced in the cells. We believe this delivery system under AMFs has remarkable potential to deliver different genes or drugs for target-specific treatment as a cancer therapy other than in siRNA delivery.

In this work, we observed the endocytosis of IO-nanocages in the osteosarcoma cell lines. The results revealed that IO-nanocages are predominantly endocytosed via a clathrin-mediated pathway in LM7 cells, while caveolae-mediated endocytosis is the most common mechanism in OS482 cells. It has been reported that the characteristics of nanoparticles and cell type influence the endocytosis of nanoparticles [35,36,37,38]. For example, carboxydextran-coated iron oxide nanoparticles of 20 nm were endocytosed in the human macrophage cells via clathrin-mediated endocytosis and FBS-coated 20 nm gold nanoparticles were mainly internalized in the human lung fibroblasts and human liver cells by clathrin-mediated endocytosis [35,47]. Moreover, the clathrin-mediated endocytosis was the main endocytic pathway for silver nanoparticles of 20 nm in human melanoma cells [48]. These results indicated that the size of the nanoparticles is one of the critical parameters for the uptake mechanism. Portilla et al. confirmed that the surface charge of the nanoparticles is another key parameter for the endocytosis mechanism [49]. Positively charged iron oxide nanoparticles were internalized through receptor-medicated endocytic pathways, and micropinocytosis in mouse macrophage cells. Neutral iron oxide nanoparticles were mainly endocytosed by caveolae-mediated endocytosis, while negatively charged iron oxide nanoparticles were internalized by clathrin and caveolae-mediated endocytosis in mouse macrophage cells. Interestingly, the internalization mechanism was different for these nanoparticles in mouse pancreatic tumor cells. Positively charged particles were mainly taken by micropinocytosis, whereas negatively charged particles were internalized through all the endocytic pathways. The mouse macrophage cells appeared to take up the neutral particles by clathrin and caveolae-mediated endocytosis.

Our finding suggested that although the same concentration of IO-nanocages and siRNAs were used, the endocytic pathway of human and mouse metastatic osteosarcoma cells was different. LM7 cells were derived from human SAOS-2 osteosarcoma cells, which were experimentally selected from the lung tissue after metastasis was observed at the seventh injection [50], whereas OS482 cells were engineered to mimic human osteosarcoma by osteoblast-restricted deletion of *p*53 and p*Rb* [51]. Although OS482 cells resemble human osteosarcoma cells in transcriptional profiles, these cells may have different genetic profiles, suggesting that internalization of siRNA delivery carriers may depend on cell type. Thus, these results suggest that not only do the characteristics of nanoparticles play an important role in the endocytosis mechanism, but also that the cell type can affect the endocytic pathways.

Similar to recent research, we also observed the effect of size, shape, and surface charge of IO-nanocages in delivering drugs. It has been previously reported that hollow cube-shaped magnetic IO-nanocages were more effective than solid spherical particles of the same diameter in delivering riluzole, a drug that inhibits the release of glutamate, in an in vivo model [18,42]. Smaller nanoparticles have been observed to avoid accumulation in the liver and spleen, allowing the dose of nanoparticles to reach other desired intervention sites [52,53,54]. Slightly negatively charged nanoparticles are better at avoiding liver uptake and preventing aggregation in blood to allow more efficient drug delivery [44,55]. IO-nanocages were demonstrated to be slightly more negatively charged compared to conventional sphere iron oxide nanoparticles when the drug was loaded. They might deliver drugs to the target sites better by bypassing hepatic clearance and preventing accumulation in the blood [42]. With these previous findings, delivery of mGluR5 siRNA using IO-nanocages as carriers in vivo has the potential to block glutamate receptor activity, which may result in inhibiting tumor proliferation. We believe that when IO-nanocages are used to deliver mGluR5 siRNAs to the targeted tumor site, AMFs application will enhance the release of mGluR5 siRNAs into the tumor site, which would result in tumor size shrinkage.

## 4. Materials and Methods

### 4.1. Iron Oxide Nanocage (IO-Nanocages) Synthesis

IO-nanocages were synthesized via Galvanic replacement reaction with a modification of a previously published method [56]. First, 1 mmol of anhydrous manganese (II) acetate, 2.5 mmol of oleylamine, and 0.5 mmol of oleic acid were added to 15 mL of p-xylene in a three-necked 50 mL flask with a reflux condenser and sonicated for 10 min. The flask was heated to 90 °C in a silicon oil bath under magnetic stirring, then 1 mL of deionized water was rapidly injected into the flask. The reaction mixture was heated at 90 °C constantly for 1.5 h, then 1 mL of 2.4 M aqueous Iron (II) perchlorate solution was added to the reaction flask. The mixture was refluxed at 90 °C for another 1.5 h to produce IO-nanocages by galvanic replacement. After cooling down for 10 min, IO-nanocages were collected by centrifugation at 3000× *g*, rinsed with ethanol, and dispersed in tetrahydrofuran (THF) solution.

### 4.2. Dihydrocaffeic Acid (DHCA)-Coated IO-Nanocages

Hydrophobic IO-nanocages in THF solution were further coated with DHCA to make them aqueous soluble, using a modified version of a previously published method [57]. Next, 30 mg of DHCA was dissolved per mL of THF in a one-neck 50 mL flask and heated to 50 °C in a silicon oil bath under magnetic stirring for 3 h. This was then cooled down to room temperature, followed by the addition of 1 mL of 0.5 M NaOH per 90 mg of DHCA to precipitate the magnetic IO-Nanocages. The precipitate was collected by centrifugation at 3000× *g*, redispersed in 2 mL of water, and the solution was placed in 10 k WMCO dialysis membrane overnight.

### 4.3. Fluorophore Conjugation to DHCA Labeled IO-Nanocages

To label DHCA-coated IO-nanocages with Cy5, first, 40 mM EDC was prepared by dissolving 3.8 mg EDC in 500 μL of H_2_O, and 50 mM NHS was prepared by dissolving 2.9 mg NHS in 500 μL of H_2_O. Then, 5 μL each of 40 mM EDC and 50 mL NHS per mg of IO-nanocages was added to a working solution of IO-nanocages in PBS. The Cy5-amine was dissolved in DMSO at 10 mg/mL, and 20 nmol of dye was added per mg of IO-nanocages and the mixture was let to react overnight. The following day, the solution was dialyzed overnight with a 3500 kD molecular weight membrane covered from light.

### 4.4. Cell Culture/Incubation

LM7 cells (human osteosarcoma cell line) [50] and OS482 cells (mouse osteosarcoma cell line) [51] were cultured in DMEM medium supplemented with 10% FBS, 100 unites/mL penicillin-100 μg/mL streptomycin, and 1% glutaMAX™ (35050061, Thermo Fisher Scientific, Waltham, MA, USA). Cells were incubated with the condition of 37 °C and 5% CO_2_ until 80–90% confluency.

### 4.5. Internalization of IO-Nanocages in LM7 and OS482 Cells

LM7 and OS482 cells (20,000 cells) were seeded in an 8-well chamber slide with 500 μL of DMEM medium supplemented with 10% FBS, 100 unit/mL penicillin-100 μg/mL streptomycin, and 1% glutaMAX™ (35050061, Thermo Fisher Scientific, Waltham, MA, USA). The next day, the media was aspirated, then supplemented with 500 μL of DMEM media followed by three washes with PBS. Then, Cy5 labeled IO-nanocages were added to those cells and incubated for 6 h at 37 °C and 5% in a CO_2_ incubator. After removing media and washing with PBS, cells were fixed in 150 μL of 4% paraformaldehyde. Cells were then blocked in buffer (5% BSA with 0.3% Triton X-100) for 1 h and incubated in primary antibody (caveolin-1; 3276, cell signaling, MA, USA. Clathrin heavy chain; 4796, cell signaling, MA, USA) overnight at 4 °C. The next day, the aspirated buffer and the cells were incubated in secondary antibody (Alexa Fluor^®^ conjugate; 4412S, cell signaling, MA, USA) for 1 h. The cells were rinsed with PBS then mounted in DAPI mounting media (p36935, Thermo Fisher Scientific, Waltham, MA, USA). Images were taken under NIKON A1 confocal microscopy and the resulting images were analyzed with the NIS element software and ImageJ.

### 4.6. Inhibit the Internalization of IO-Nanocages in LM7 and OS482 Cells

LM7/OS482 cells (20,000 cells) were seeded in an 8-well chamber slide with 500 μL of DMEM medium supplemented with 10% FBS, 100 unit/mL penicillin-100 μg/mL streptomycin, and 1% glutaMAX™ (35050061, Thermo Fisher Scientific, Waltham, MA, USA). The next day, the media was aspirated and 500 μL of serum free DMEM media was added and incubated for 1 h. Following this, the media was aspirated and cells were pretreated with pitstop2 (25 μM) [39] for 15 min and Nystatin (50 μg/mL) [40,41] for 30 min at 37 °C. Then, the solution was removed, and the cells were washed three times with PBS. Further steps were the same as described in the internalization experiment above.

### 4.7. Application of Alternating Magnetic Fields (AMFs) to Treat LM7 and OS482 Cells

After the siRNA-loaded IO-nanocages were taken up by LM7 and OS482 cells in 35 × 10 mm dishes for 18 h, they were placed in a magnetic field coil which generates 445 kHz and the AMFs were applied for 5 min, followed by an additional 6 h of incubation. Magnetic fields are generated inside the coil when electric currents are applied to the coil, and the magnitude of the magnetic field depends on the input electric potential. The magnetic strength of the coil depends on the number of loops of coil in addition to the input voltage. After a total of 24 h of treatment, cells were ready for conducting further experiments.

### 4.8. Luciferase Assay

Pierce^®^ Firefly Luciferase Glow Assay Kit (16176, Thermo Fisher Scientific, Waltham, MA, USA) was used to measure the bioluminescence of the luciferase expression in LM7/OS482 cells. Then, 750 nM of firefly luciferase siRNAs (AM4629, Thermo Fisher Scientific, Waltham, MA, USA) were incorporated into IO-nanocages (9 × 10^11^ particles) overnight in Opti-MEM™ medium (11058021, Thermo Fisher Scientific, Waltham, MA, USA). Following this, 0.2 × 10^6^ cells were plated in 35 × 10 mm cell culture dishes simultaneously and incubated overnight at 37 °C and 5% CO_2_. The next day, the cells were treated with a complex of siRNA-loaded IO-nanocages for a total of 24 h, then luciferase assay was performed. After incubation for a total 24 h, the media were aspirated from the cells and rinsed with PBS, then 400 µL of 1X Luciferase cell-lysis buffer was added to both the absence and presence of AMFs application experimental groups. These plates were placed on the moderate speed shaker for 15 min to lysate the cells, and 10–20 µL of the samples were added to a white opaque 96-well plate. Then, 50 µL of working solution was added to each well. These samples were left for another 10 min for signal stabilization at room temperature, and the bioluminescence was measured.

### 4.9. Quantitative RT-PCR

Total RNA was extracted using RNeasy^®^ mini kit (74104, Qiagen, Hilden, Germany) from LM7 and OS482 cells (0.2 × 10^6^ cells) after 24 h of treatment with human/mouse mGluR5 siRNA-loaded IO-nanocages in the presence or absence of AMF, following the manufacturer’s manual. Total RNA was eluted in 50 μL of RNase-free water and 1 μg of total RNA was converted to cDNA using the QuantiTect^®^ reverse transcription kit (205311, Qiagen, Hilden, Germany) following the manufacturer’s instructions. To quantify the mRNA expression level in LM7 and OS482 cells, the cDNA template was mixed with SYBR^®^ Green (Thermo Fisher Scientific, Waltham, MA, USA) and 0.4 μM of each forward and reverse primer, which was subjected to a total volume of 25 μL. The cycle setting was 95 °C for 10 mins and 40 cycles (15 s denaturation at 95 °C, 1 min annealing at 60 °C, and 1minute extension at 72 °C). Primer sequences used for the LM7 human Osteosarcoma cell line were as follows: Human mGluR5 (5′-TGGAGATACGATCCTATTCG-3′ and 5′-CCAAGGCAGGCAAACACCAC-3′) and human β-actin (5′-CCTTCCTGGGCATGGAGT -3′ and 5′-AGGAGCAATGATCTTGAT-3′). Primer sequences used for OS482 mouse osteosarcoma cell line were the following: mouse mGluR5 (5′-ATCTGCCTGGTTACTTGTG-3′ and 5′-GCAATACGGTTGGTCTTCG-3′) and mouse GAPDH (5′-TGCACCACCAACTGCTTAGC-3′ and 5′-GGCATGGACTGTGGTCATGAG-3′). The relative cycle threshold (C_t_) values were calculated for each sample by normalizing C_t_ of mGluR5 to C_t_ of β-actin in LM7 cell samples and normalizing C_t_ of mGluR5 to C_t_ GAPDH in OS482 cell samples.

### 4.10. MTT Assay

LM7 (50,000 cells) and OS482 (40,000 cells) cells were seeded in 35 × 10 mm dishes overnight and treated with human mGluR5/ mouse mGluR5 siRNA-loaded IO-nanocages in the presence or absence of AMF for a total of 24 h. After the treatment, 10% of the MTT (5 mg/mL) solution was added to the media in each dish. Cells were placed for approximately 3 h in the incubator (5% CO_2_ at 37 °C), then the media were removed. Furthermore, 100% DMSO was added to the cells and incubated for 5 min at room temperature. Then, 250 μL of the solution was transferred to a 96-well plate (triplet) and read at 570 nm immediately.

### 4.11. Clonogenic Assay

The Clonogenic assay was performed using the method described previously with modifications [58]. Briefly, 300 cells of LM7 and OS482 were seeded in 35 × 10 mm dishes and grown for 3 days. Then, cells were treated with human mGluR5/ mouse mGluR5 siRNA-loaded IO-nanocages. For the groups with AMFs application, AMFs was applied for 5 min after 18 h of treatment and incubated for an additional 6 h, making it a total of 24 h of treatment. For the groups without AMFs application, cells were incubated for 24 h and collected for further experiments. After 24 h of treatment, cells were fixed in 4% paraformaldehyde and stained with 0.05% Cresyl violet. Three independent trials were conducted and an average number of colonies were obtained.

### 4.12. Statistical Analysis

The significance of the experiment weas determined by one-way ANOVA using SPSS software and each experiment was conducted at least three times. The schematic representation in Figure 2A and Figure 5 was made using Biorender.com.

## 5. Conclusions

In conclusion, we demonstrated that mGluR5 siRNA was delivered by IO-nanocages and reduced mGluR5 expression in both human and mouse osteosarcoma when AMF was applied. When 100 nM of mGluR5 siRNA was delivered with IO-nanocages (the number of particles = 9 × 10^11^) in 5 min of AMFs, this method worked the best to enhance siRNA delivery efficiency. Moreover, we confirmed that the silencing of mGluR5 expression in the cells decreased the proliferation of cells and inhibited the formation of colonies, suggesting that mGluR5 plays a pivotal role in glutamate-dependent osteosarcoma proliferation. Since this delivery methodology worked efficiently for siRNA delivery, this model can potentially be applied to deliver various siRNAs or drugs for cancer therapy. Our findings revealed successful transfection efficiency using the IO-nanocages delivery methodology, resulting in the inhibition of cell growth, which may lead to its clinical improvement as a treatment.

## 6. Patents

1. Matsui, H.; Paragodaarachchi, A.; Fang, J. “Method of Forming Inorganic Nanocages”. U.S. Patent: 113,251,12.

## Data Availability

The raw/processed data of delivery system model are available upon request (hmatsui@hunter.cuny.edu) and application works are available upon request from the corresponding author. All remaining data are included in manuscript.
